# Systemic overexpression of C-C motif chemokine ligand 2 promotes metabolic dysregulation and premature death in mice with accelerated aging

**DOI:** 10.18632/aging.104154

**Published:** 2020-10-26

**Authors:** Fedra Luciano-Mateo, Noemí Cabré, Gerard Baiges-Gaya, Salvador Fernández-Arroyo, Anna Hernández-Aguilera, Elisabet Elisabet Rodríguez-Tomàs, Meritxell Arenas, Jordi Camps, Javier A. Menéndez, Jorge Joven

**Affiliations:** 1 Universitat Rovira i Virgili, Department of Medicine and Surgery, Reus 43201, Spain; 2 Unitat de Recerca Biomèdica, Hospital Universitari Sant Joan, Institut d’Investigació Sanitària Pere Virgili, Universitat Rovira i Virgili, Reus 43201, Spain; 3 Department of Radiation Oncology, Hospital Universitari Sant Joan, Institut d’Investigació Sanitària Pere Virgili, Universitat Rovira i Virgili, Reus 43204, Spain; 4 Program Against Cancer Therapeutic Resistance (ProCURE), Metabolism and Cancer Group, Catalan Institute of Oncology, Girona 17007, Spain; 5 Girona Biomedical Research Institute (IDIBGI), Girona 17190, Spain; 6 The Campus of International Excellence Southern Catalonia, Tarragona 43003, Spain

**Keywords:** C-C motif chemokine ligand 2, energy metabolism, one-carbon metabolism, progeria

## Abstract

Injection of tissues with senescent cells induces changes that mimic aging, and this process is delayed in mice engineered to eliminate senescent cells, which secrete proinflammatory cytokines, including C-C motif chemokine ligand 2 (*Ccl2*). Circulating levels of *Ccl2* correlate with age, but the impact of *Ccl2* on tissue homeostasis has not been established. We generated an experimental model by crossbreeding mice overexpressing *Ccl2* with progeroid mice bearing a mutation in the lamin A (*Lmna)* gene. Wild-type animals and progeroid mice that do not overexpress *Ccl2* were used as controls. *Ccl2* overexpression decreased the lifespan of the progeroid mice and induced the dysregulation of glycolysis, the citric acid cycle and one-carbon metabolism in skeletal muscle, driving dynamic changes in energy metabolism and DNA methylation. This impact on cellular bioenergetics was associated with mitochondrial alterations and affected cellular metabolism, autophagy and protein synthesis through AMPK/mTOR pathways. The data revealed the ability of *Ccl2* to promote death in mice with accelerated aging, which supports its putative use as a biomarker of an increased senescent cell burden and for the assessment of the efficacy of interventions aimed at extending healthy aging.

## INTRODUCTION

Mechanisms leading to accelerated aging are not completely understood but might converge at points of inflammation and undesirable metabolic alterations [1, 2]. Indeed, the increased incidence of chronic noncommunicable diseases during aging is closely related to the progressive decline of immune and metabolic functions. Understanding how and why we age and how to manage immunometabolic alterations in elderly individuals are fundamental challenges to the field of medicine [2, 3]. Metformin, a drug that may potentially modulate the interplay between the metabolic processes associated with inflammation and cellular senescence, has been chosen to explore pharmacologic effects that target aging-related pathways [4–6]. Clinical trials aimed to test potential anti-aging treatments confront multiple limitations because of the extensive time between the interventions and the expected outcomes. No easily accessible biomarkers enable the prediction of functional capabilities, establishment of surrogate end-points, assessment of changes in the processes associated with aging or measurements of biological age [7–9]. A promising exception might be a multitissue DNA methylation-based age estimator applicable throughout the entire age spectrum [10, 11]. Its use in epidemiological studies indicates that epigenetic markers of aging acceleration appear to be closely associated with metabolic and inflammatory biomarkers [12]. Senescent cells in a permanent state of cycle arrest progressively accumulate during aging and contribute to the release of components associated with the senescence-associated secretory phenotype (SASP), which may partially explain the increased metabolic inflammation observed during the aging process [13–17]. These findings support the putative relevance of an abnormal secretion of proinflammatory cytokines and C-C motif chemokine ligand 2 (*Ccl2*) during aging. High levels of circulating *Ccl2* are common in diseases associated with tissue injury and have also been shown to correlate with biological age. Such associations likely reflect an increased burden of senescent cells and have been proposed as potential tools to measure the effect of interventions aimed at extending healthy aging [18, 19].

To investigate the effects of the systemic increase in *Ccl2* expression on progeria-induced metabolic derangement and lifespan, we generated an experimental model by crossbreeding mice that overexpress *Ccl2* [20] with mice bearing a mutation in exon 11 of the lamin A gene (*Lmna*) to obtain offspring that recapitulate most of the clinical features of Hutchinson-Gilford progeria syndrome in humans (HPGS) [21–23]. Histological evidence obtained from these models and the particular relevance of muscle-wasting disorders in the aging population suggest the importance of specifically exploring molecular processes associated with aging in skeletal muscle, including energy and one-carbon (1-C) metabolism alterations or mitochondrial dysfunction, and the metabolic pathways associated with cell survival [22, 23]. Our findings suggest that *Ccl2* is involved in the aging process and should be considered in the assessment of immunometabolic dysfunction in age-related diseases.

## RESULTS

### *Ccl2* overexpression reduced the lifespan of mice with accelerated aging

**Figure 1:**
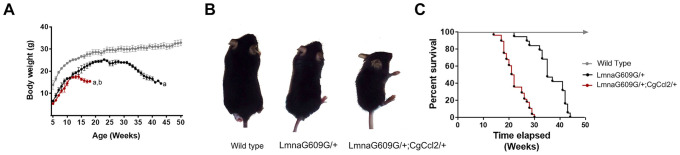
***Ccl2* significantly reduced the lifespan of mice with accelerated aging.** *Ccl2* overexpression was critical for (**A**) the differential progression in body weight with age, (**B**) development of features of accelerated aging, as shown in the representative photographs of mice at 20 weeks of age, and (**C**) reduced lifespan, as shown by Kaplan-Meier survival plots from the wild-type (n=20), LMNAG609G/+ (*n=28)* and LMNAG609G/+;CGCCL2+/- (n=34) mice. LMNAG609G/+;CGCCL2+/- and LMNAG609G/+ denote progeroid mice overexpressing and not overexpressing Ccl2, respectively.

**Figure 2:**
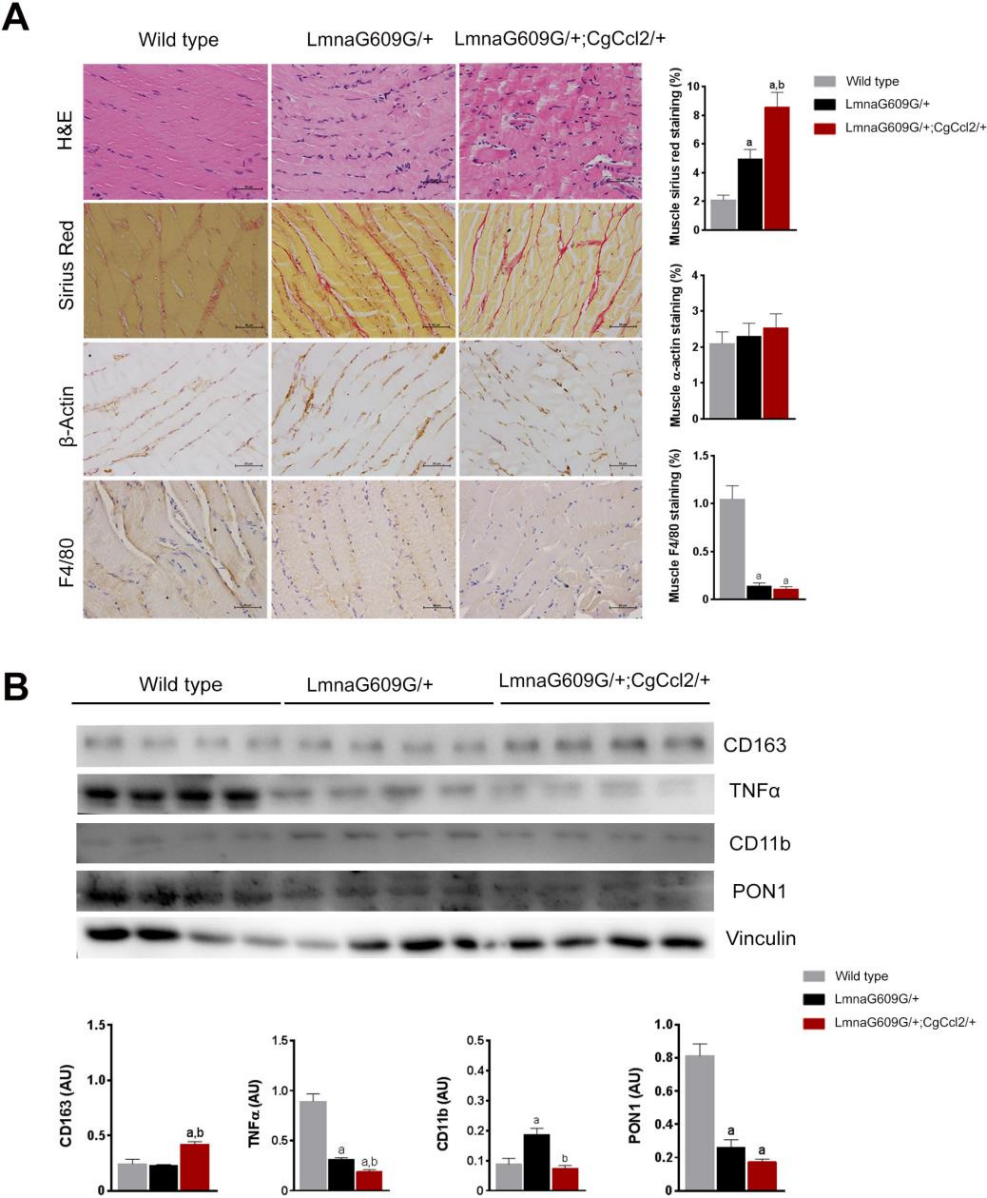
**Histological phenotypes were altered in the muscles of aged mice.** The structural changes in quadriceps muscles are depicted in representative micrographs of tissue stained with hematoxylin and eosin and Sirius red staining and determined by immunohistochemical analysis of β-actin and F4/80 cells. *Ccl2* overexpression accelerated the presence of histological alterations (**A**). Fibrosis was a prominent feature in the progeroid mice, but the relative amount of actin-stained cells was similar among strains, despite major differences in muscle weight. Interestingly, the number of F4/80-stained cells was significantly lower in the progeroid mice. We then used immunoblotting to examine (**B**) the expression of the markers of macrophage polarization, including cluster of differentiation (CD) 11 and 163, and tumor necrosis factor (TNFα) to indicate the relative anti-inflammatory activity, which was accompanied by lower expression of the major antioxidant enzyme paraoxonase 1 (PON1) in both progeroid strains. The LMNAG609G/+;CGCCL2+/- mice were compared with wild-type mice, as depicted by ^a^ p<0.05, and with LA ^*+/-*^ mice, as depicted by ^b^ p<0.05, according to the Mann–Whitney U tests (n=15 for each strain). LMNAG609G/+;CGCCL2+/- and LMNAG609G/+ denote progeroid mice overexpressing and not overexpressing Ccl2, respectively.

The increase in body weight significantly differed among strains, and *Ccl2* overexpression was associated with different body weight changes, significant impairment in fertility, earlier-than-normal dermal thinning and considerable loss of muscle and fat. Mice with a single mutation in lamin A (LMNAG609G/+) gradually lost weight with respect to the weight of the wild-type mice from 25 to 32 weeks and the mice with a mutation in lamin A and overexpressing Ccl2 (LMNAG609G/+;CGCCL2+/-) from 12 to 14 weeks (Figure 1A). The progeroid strains also progressively diverged in terms of the development of features of accelerated aging, including growth rate, lipodystrophy and lordokyphosis (Figure 1B). The median lifespan was significantly reduced (33%) in the mice with overexpressed *Ccl2* (45 weeks for the LMNAG609G/+ mice and 30 weeks for the LMNAG609G/+;CGCCL2+/- mice) (Figure 1C). Degenerative cardiovascular changes, cachexia and sarcopenia also developed earlier in the LMNAG609G/+;CGCCL2+/- mice than in LMNAG609G/+ mice. The progeroid strains showed major histological alterations in the aorta and femoral arteries with respect to the controls. The extent of arterial fibrosis was greater in the LMNAG609G/+;CGCCL2+/- and LMNAG609G/+ mice than in the controls. Although the expression of actin and the number of stained macrophages were significantly higher in the mice with overexpressing *Ccl2*, the differences in fibrosis between the progeroid strains did not reach statistical significance (Supplementary Figure 1). The histological structure of the cardiac muscles in the progeroid mice was also significantly altered, and the changes were immediately apparent with routine staining procedures. Increased fibrosis of cardiac muscles was the most prominent feature caused by *Ccl2* overexpression (Supplementary Figure 2). Similarly, the generalized loss of fat depots and the extent of the abnormal structure of brown and epididymal white adipose tissue were significantly greater in the mice overexpressing *Ccl2*. In the tissues from both progeroid mouse strains, the adipocyte area was lower with higher expression of uncoupling protein one and an increased number of stained F4/80 cells compared to these levels in the controls. All these features and fibrosis were significantly affected by *Ccl2* overexpression (Supplementary Figure 3). The sarcopenia and cachexia causing the skeletal muscle loss in the genetically modified mice were significantly altered by *Ccl2* overexpression. The histological changes in both models of accelerated aging showed that fibrosis was significantly enhanced, but the response against oxidation was decreased, and the number of anti-inflammatory macrophages in muscle was significantly lower compared to those of the controls (Figure 2). The data indicate that *Ccl2* overexpression promoted death in mice with accelerated aging and confirmed the role of *Ccl2* as a fibrosis-inducing agent with a negative impact on biological outcomes associated with aging and/or longevity. A caveat of this set of experiments suggests that the ages at which the animals were studied differed for each experimental group (38-40 weeks for WT, 32 weeks for LMNAG609G/+, 18-22 weeks for LMNAG609G/+;CGCCL2+/-), since we decided to sacrifice the animals when they presented a similar degree of senescence, according to the criteria described in Materials and Methods. The age difference could explain, at least partially, the difference in the results that we observed in the arteries.

### *Ccl2* overexpression drove specific signatures of metabolic dysregulation in the skeletal muscles of the progeroid mice

**Figure 3:**
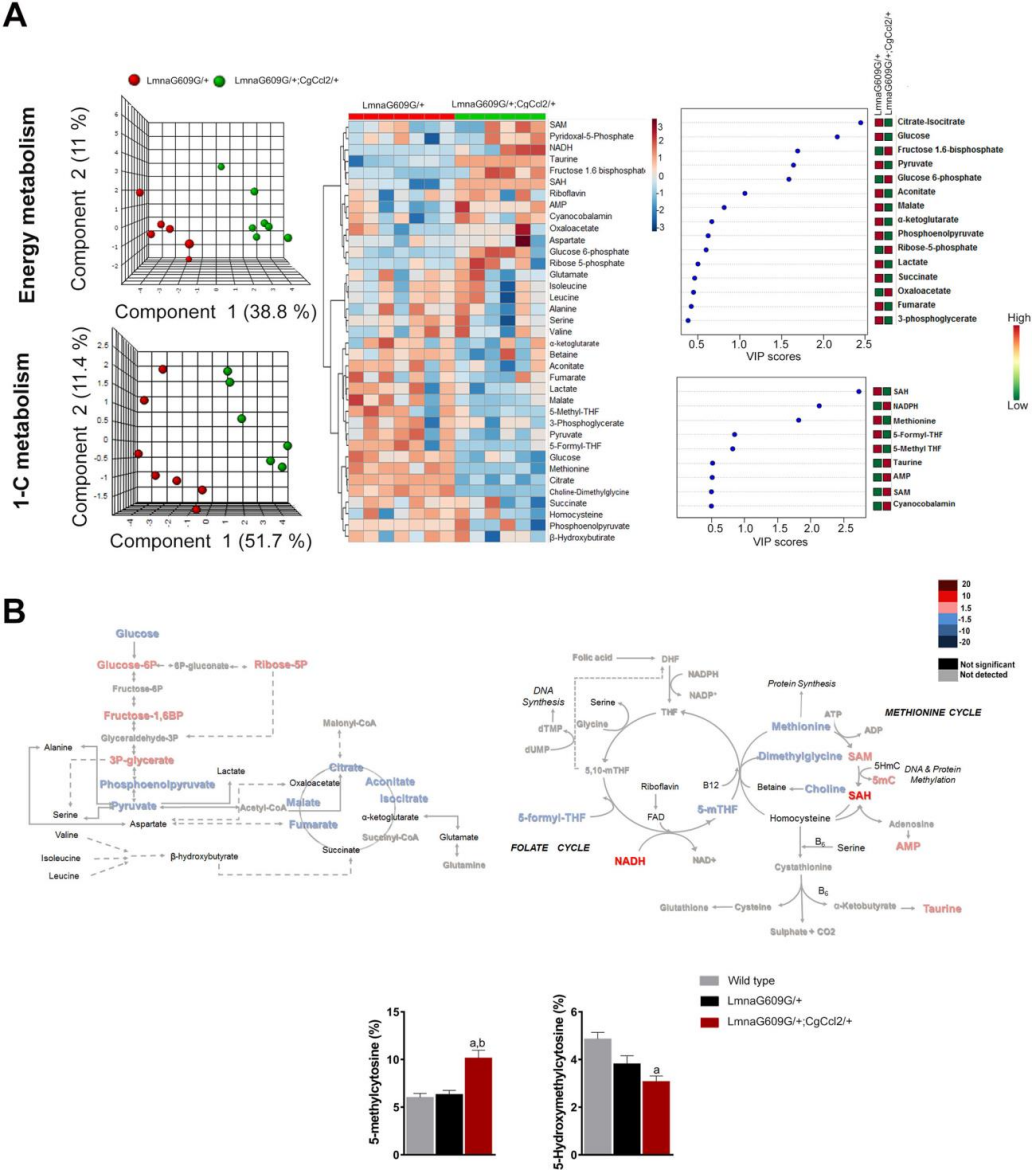
**The impact of *Ccl2* overexpression on the metabolic pathways associated with energy and 1-C metabolism.** Metabolomic analysis of skeletal muscle from progeroid mice with (LMNAG609G/+;CGCCL2+/-) or without (LMNAG609G/+) increased *Ccl2* levels revealed the metabolic impact of *Ccl2* in accelerated aging. (**A**) From left to right, partial least square discriminant analysis (PLSDA), heatmap with hierarchical clustering and random forest analysis indicating that *Ccl2* drove changes in energy and 1-C metabolism in the quadriceps muscles of the genetically modified mice. The levels of metabolites were distinct in both strains, and the variables of highest importance, according to the projection scores, were glucose, citrate, SAH and methionine. (**B**) Comparison of the levels of metabolite abundance from glycolysis and the CAC cycle in the quadriceps muscles were assessed by fold changes, as indicated in the legend, suggesting that Ccl2 decreased mitochondrial oxidative metabolism. (C) Dysregulation in the methionine cycle was, at least partially, the cause of the increased DNA methylcytosine levels in the LMNAG609G/+;CGCCL2+/- mice with respect to the controls and LMNAG609G/+ mice. Values are shown as the means ± SEM; ^a^p< 0.05 with respect to the wild-type mice and ^b^ p<0.05 with respect to the LMNAG609G/+ mice, according to the Mann–Whitney U tests.

To better understand the changes induced by lamin A mutation, we first performed a metabolomic analysis in muscle from fasted LMNAG609G/+ and WT mice. We selected this tissue due to its important implications in aging development. Metabolites from energy balancing and 1-C metabolism were quantitatively assessed, and normalized concentrations from all strains are depicted in Supplementary Tables 1, 2. The sets of metabolites studied demonstrated a clear distinction between LMNAG609G/+ and the controls. The partial least square discriminant analysis and the heatmap showed a clear distinction between animals from both groups (Supplementary Figure 4). The examination of individual metabolites showed that the LMNAG609G/+ animals had increased concentrations of compounds involved in energy metabolism. Notably, the increase in the concentrations of metabolites related to glycolysis, the analyzed amino acids, and the anaplerotic reactions in the CAC was important. 1-C metabolism was also altered in these mice, and the methionine level was significantly increased with a decrease in 5-methyl-tetrahydrofolate (5-methyl-THF), S-adenosylmethionine (SAM) and S-adenosylhomocysteine (SAH) levels, although we did not observe any significant alterations in DNA methylation (Figure 3B). These data likely indicate a combined defective function in the folate and methionine cycles.

The relative impact on the concentrations of these metabolites was different in the LMNAG609G/+;CGCCL2+/- mice than it was in the controls (Supplementary Figure 5). The main alterations observed were an increase in branched-chain amino acids (valine, isoleucine and leucine), β-hydroxybutyrate, fructose 1,6-bisphosphate, glucose 6-phosphate and ribose-5-phosphate concentrations, together with a depletion in 3-phosphoglycerate and citrate-isocitrate. Regarding 1-C metabolism, we observed an increase in riboflavin and methionine levels and a decrease in 5-methyl-THF, S-adenosylmethionine (SAM) and SAH. These alterations in 1-C metabolism induced an increase in the percentage of 5-methylcytosine (5-mC), which is a marker of DNA methylation. Interestingly, with respect to the controls, in both progeroid strains, the accumulation of the amino acids valine, leucine and isoleucine was significant.

We finally compared the metabolic alterations of LMNAG609G/+-;CGCCL2+/- with those of LMNAG609G/+, and we found a significant impact on the amount of metabolites in skeletal muscle that distinguished the strains, according to the results of the principal component analysis (Figure 3). Regarding energy metabolism, the LMNAG609G/+;CGCCL2+/-^-^ mice showed alterations in the glucose and citric acid cycles without significant alterations in amino acid concentrations with respect to the LMNAG609G/+ mice. In relation to 1-C metabolism, the LMNAG609G/+;CGCCL2+/- mice showed the downregulation of methionine, choline-dimethylglycine, 5 methyl-THF and 5 formyl-THF. These alterations may have been a consequence of the increase in SAM, SAH and AMP levels that we found in these animals. One of the most important consequences of SAM to SAH alterations was the increase in 5-mC. These findings might be relevant because they strongly suggest alterations in muscle DNA methylation and demethylation. DNA methyltransferases use SAM as a substrate to produce SAH; DNA demethylation depends on α-ketoglutarate and other metabolites from the CAC [24]. *Ccl2* overexpression significantly increased the methylation of cytosine bases in muscle DNA and significantly decreased 5-hmC levels with respect to both the LMNAG609G/+ mice and controls (Figure 3C). Taken together, our data indicate that *Ccl2* overexpression in the experimental model of progeria alters energy and 1-C metabolism and drives dynamic changes in DNA methylation. The role of these epigenetic marks remains to be ascertained.

### *Ccl2* overexpression disrupted mitochondrial dynamics and altered AMPK/m-TOR-driven pathways in the progeroid mice

Our data might suggest key roles of mitochondrial metabolites in pathological aging. Although the mechanisms are uncertain, *Ccl2* overexpression interferes with mitochondrial function, particularly in the oxidative phosphorylation system (OXPHOS) and the production of ATP and reactive oxygen species (ROS) [25]. Indeed, our group previously reported that transgenic mice with a WT background that overexpress Ccl2 show alterations in mitochondrial morphology; therefore, the matrix is less electron-dense. We also found altered fusion dynamics. In transgenic mice fed a chow diet, a tendency of the unbalanced dynamics shifted towards mitochondrial fusion, but dietary manipulation elicited a significant shift towards fission. These putative mechanisms of the altered metabolism seem to be related to the autophagic response [20].

**Figure 4:**
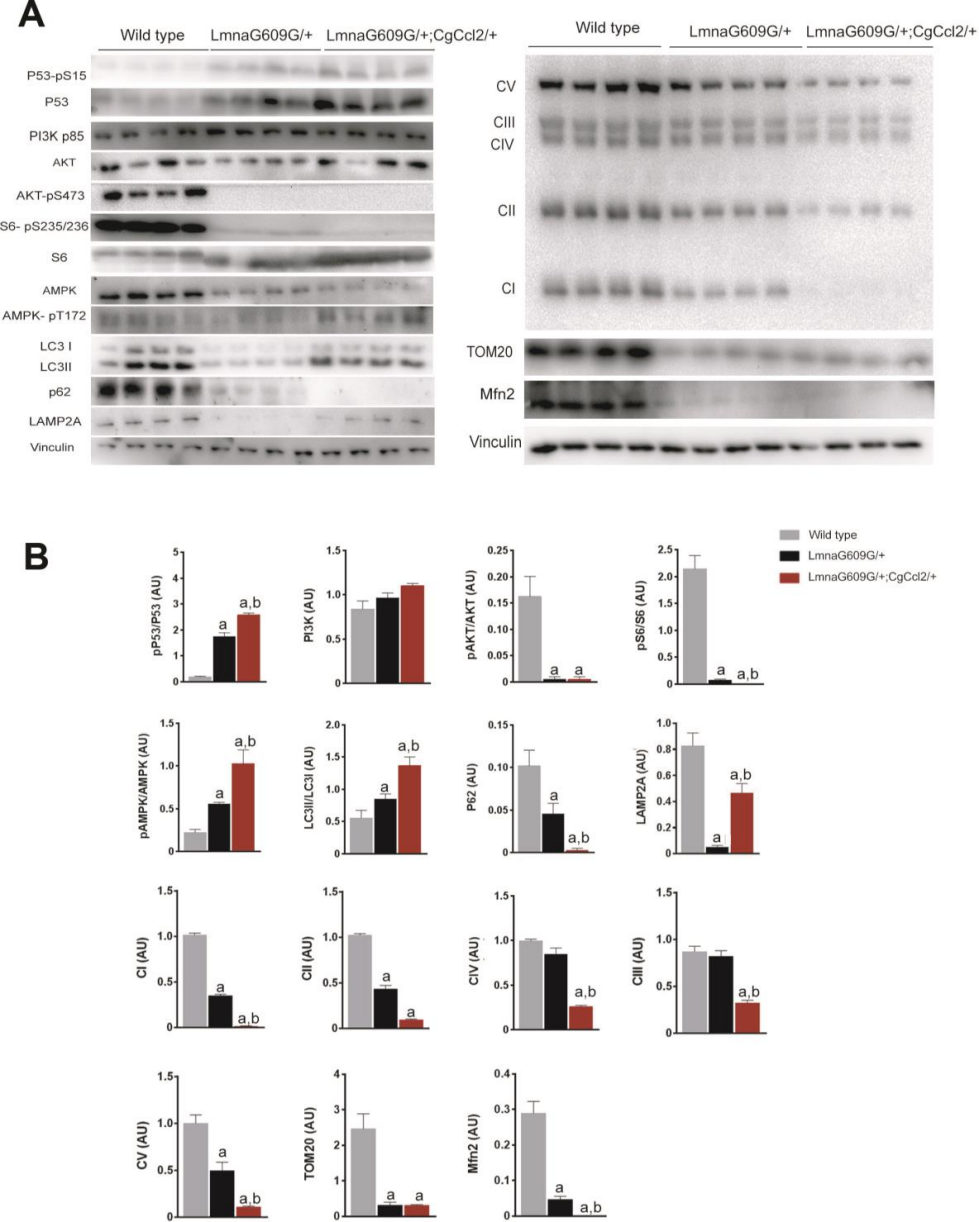
**Ccl2 overexpression significantly altered the expression of the complexes related to oxidative phosphorylation and the relevant metabolic signaling pathways in the quadriceps muscle.** Ccl2 overexpression exacerbated the metabolic reduction, mainly in mitochondria, (A) of the mice with accelerated aging and altered (B) the functioning of AMPK-mTOR-driven pathways. LMNAG609G/+;CGCCL2+/- and LMNAG609G/+ denote progeroid mice overexpressing and not overexpressing Ccl2, respectively. Values are shown as the means ± SEM; ^a^ p< 0.05 with respect to the wild-type mice and ^b^ p<0.05 with respect to the LMNAG609G/+ mice according to the Mann–Whitney U tests. Acronyms in (A) denote complex I or NADH/ubiquinone oxidoreductase, complex II or succinate dehydrogenase, complex III or cytochrome C reductase, complex IV or cytochrome C oxidase, complex V or ATP synthase, translocase of outer membrane (TOM20), and mitofusin 2 (Mfn2); acronyms in (B) phospho-tumor suppressor p53 (p53-pS15), tumor suppressor p53 (P53), phosphoinositide 3-kinase (PI3K), protein kinase B (AKT) and phospho-protein kinase B (AKT-pS473), phospho-ribosomal protein S6 (S6-pS235/236), ribosomal protein S6 (S6), AMPK activated protein kinase (AMPK), phospho-AMPK activated protein kinase (AMPK pT172), microtubule-associated proteins 1A/1B light chain 3B (LC3), p62 adaptor protein or sequestosome 1, and lysosome-associated membrane protein 2 (LAMP2A).

We therefore explored the expression of the mitochondrial respiratory complexes in skeletal muscle. Complexes I and II are major players in mitochondrial signaling and ROS generation, and their expression was significantly decreased in the progeroid mice compared with that of the controls. The expression levels, especially of complex I, were lower in the LMNAG609G/+;CGCCL2+/- mice than in the LMNAG609G/+ mice. Additionally, complexes III and IV appeared normal in the muscles of the LMNAG609G/+ mice with respect to the muscles of the controls, but *Ccl2* overexpression in the LMNAG609G/+;CGCCL2+/- mice significantly decreased complex expression. Finally, the expression of complex V (ATP synthase) in the muscles of the LMNAG609G/+ mice was significantly decreased, and this effect was significantly more prominent in the LMNAG609G/+;CGCCL2+/- mice. In addition, the protein concentrations of translocase of outer membrane 20 (TOM20) and mitofusin 2 (MFN2) in the muscles of the progeroid mice were decreased, indicating alterations in the proper formation of the mitochondrial network (Figure 4A).

*Ccl2* overexpression had a negative impact on the cellular bioenergetics in progeroid skeletal muscle, and this negative effect was associated with reduced efficiency of mTOR-related homeostatic mechanisms. In the muscles of the LMNAG609G/+ mice, the expression and phosphorylation of p53 was increased with respect to that of the controls, and the level of p53 activation in the LMNAG609G/+;CGCCL2+/- mice was significantly increased with respect to that in the LMNAG609G/+ mice, which likely indicates the triggering of aging through a p53-mediated transcriptional program. As p53 is a major factor in the cell cycle and proliferation and because senescent cells may be more or less metabolically active, the differential response may be the result of the differences in the expression and activation of related molecules, most likely those associated with the mechanistic target of rapamycin (mTOR). We explored major upstream and downstream pathway players and found that the phosphoinositide 3-kinase (PI3K)/AKT (also known as protein kinase B) pathway, with critical roles in regulating diverse cellular functions, was abnormal. PI3K and AKT expression was not affected in the cells of either the progeroid or the *Ccl2*-overexpressing strains. However, AKT was not phosphorylated at S473 in the muscle cells of either progeroid mouse strain, and S6 phosphorylation was significantly decreased in the LMNAG609G/+ mice and undetectable in the LMNAG609G/+;CGCCL2+/- mice, indicating that mTOR signaling is significantly inhibited in these models of accelerated aging. AMP-activated protein kinase (AMPK) expression was significantly lower in the cells of the genetically modified mice, but its relative phosphorylation was significantly higher. The effect was more pronounced in the LMNAG609G/+;CGCCL2+/- mice than in LMNAG609G/+ mice. The cells overexpressing *Ccl2* in the LMNAG609G/+;CGCCL2+/- mice apparently favored autophagy with respect to the cells in the LMNAG609G/+ mice, as indicated by a higher LC3II/I ratio. However, the expression levels of the lysosomal membrane proteins LAMP2A and p62 were decreased in the progeroid mice, suggesting the involvement of chaperone-mediated autophagy in aging and the ability of *Ccl2* to interfere in this process (Figure 4B). However, we cannot verify that CCL2 decreases ATP levels AMPK or, in contrast, AMPK is activated because of other changes, such as the upregulation of Ca^2+^ levels.

## DISCUSSION

The activation of the immune machinery critical for chronic inflammation has been associated with age-related diseases and the process of aging [2, 3, 26]. There is no clear understanding of the mechanisms that initiate this relationship, but several common molecular pathways have been identified through their characteristic increase in senescent cells during aging [27]. Senescent cells secrete factors that include chemokines, which have complex effects on the trafficking of immune cells [13, 28]. Epidemiological studies have suggested that chemokine levels are increased in aged individuals, particularly Ccl2, with or without metabolic alterations, and *in vitro*, they appear to confer senescence even to neighboring normal cells in an autocrine and paracrine fashion [18, 29, 30]. The ubiquitous expression of *Ccl2* in aged tissues suggests a potentially important role, but the analysis and interpretation remain complicated by the joint expression of functional and atypical receptors and antioxidant enzymes [31]. We previously studied the effect of only *Ccl2* overexpression in mice with a WT background [20]. The mice resulting from the targeted mutation were viable, fertile and normal in size and weight and showed no apparent behavioral or reproductive defects. We also found that the mutant mice died prematurely between 10 and 14 months when given a high-fat diet but not when given a normal diet. Furthermore, we also reported that *Ccl2*-deficient mice were protected from oxidative stress, inflammation and metabolic disturbances and had a normal lifespan [15]. To investigate the prosenescence effects of *Ccl2* in accelerated aging at the organismal level, we developed an experimental model that combines progeria induced by a mutation in the *Lmna* gene and systemic *Ccl2* overexpression. The results indicated that excessive *Ccl2* promoted the death of the mice with progeria. *Ccl2* overexpression reduced the average mouse lifespan, accelerated the development of premature degenerative changes and caused metabolic dysregulation. The mechanisms are likely multifaceted but appear to be independent, at least partially, from their roles as chemoattractants because macrophages did not significantly accumulate in all the tissues. The cause of death was unclear, but *Ccl2* overexpression was a significant promoter of fibrosis in all the tissues.

Dysregulation of certain metabolites may lead to altered cell function, including immune cells, in a model characterized by increased accumulation of progerin [23]. Progerin is a mutant protein in the nuclear lamina encoded by the *Lmna* gene and has been linked to multiple clinical pathologies, including the skeletal myopathies and premature aging observed in HPGS [32]. In mice, the conditional overexpression of progerin in muscle induced multiple metabolic defects and premature death, indicating an intrinsic effect of progerin on energy metabolism and aging [33, 34]. Muscle metabolites from energy and 1-C metabolism revealed major alterations in energy expenditure and mitochondrial dynamics in the *Lmna*^+/-^ mice, and these outcomes were negatively affected by *Ccl2* overexpression. Specifically, *Ccl2* contributed to the mitochondrial dysfunction and impairment of cellular bioenergetics in the muscles of the progeroid mice with complex metabolic alterations, which may have affected cellular survival, autophagy and protein synthesis, possibly contributing to the histological alterations and decreased mouse lifespan. The molecular damage caused by increases in progerin [35] emerged when the cumulative damage caused by accelerated aging was not ameliorated by compensating homeostatic mechanisms. The impact of *Ccl2* overexpression likely included the impairment of these compensatory mechanisms and, in particular, a reduction in the adaptation of mitochondrial function in the mice with accelerated aging. It was noticeable that progeria and *Ccl2* overexpression increased methionine levels in the muscles and altered the DNA methylation status, likely with associated epigenetic changes. Our experimental model combined the impairment of mitochondrial function and dysregulation in methionine metabolism that altered SAM and SAH levels [36]. Interestingly, high methionine levels in human diseases are associated with profound alterations in muscle [37], and dietary methionine restriction, which in progeroid mice decreased inflammation and enhanced mitochondrial activity, has been associated with increased lifespan [38].

One of the most characterized senescence markers is p53, which we found to be increased in both progeroid mouse strains. The interactions between p53 and mTORC1 are regulated by several factors. For instance, it has been demonstrated that p53 induces the direct or indirect activation of AMPK, which is considered to be one of the most important inhibitors of mTORC1. Moreover, p53 also induces the PTEN/PI3K/AKT pathway to reduce mTORC1 activity [39]. This metabolic reprogramming causes systemic alterations in cells, such as upregulated autophagy. Autophagy promotes the recycling of damaged organelles in response to metabolic alterations or nutrient depletion [40–42] and is considered to be one of the most important anti-aging mechanisms of cells. Interestingly, we observed an increase in macroautophagy in both groups of progeroid mice despite their accelerated aging. Similar results were observed using mouse models of progeria [43, 44]. We suggest that macroautophagy activation is an adaptive response to the metabolic stress observed in these mice. Moreover, upregulated macroautophagy may be a compensatory mechanism to attenuate the chaperone-mediated autophagy impairment (CMA) observed in both groups. The downregulation of CMA is associated with loss of protein degradation machinery [45], a finding that may explain the increase in amino acid concentrations observed in the LMNAG609G/+;CGCCL2+/- and LMNAG609G/+ mice. A decline in protein homeostasis is closely related to aging progression and degenerative diseases such as Alzheimer’s disease, Parkinson’s disease, Huntington’s disease and cancer [45]. Mitochondrial function, autophagy, and glycolysis are closely linked and related to each other in a complex manner that is not yet well understood. Our results show activation of glycolysis in mice that overexpress Ccl2 despite the inactivation of the mTORC1 system. We do not know the alternative mechanism that explains this phenomenon, but it is known that p53 regulates many aspects of metabolism, such as glycolysis, mitochondrial oxidative phosphorylation and glutaminolysis, and we think that this pathway should be explored in subsequent studies.

In conclusion, our findings indicate that *Ccl2* signaling promotes death and negatively impacts the energetic balance and muscle homeostasis in mice with lamin A mutations and accelerated aging. In this study, we describe an animal model that combines the overexpression of *Ccl2* in mice with a progeroid background, and we report that these animals present important metabolic alterations in muscle tissue. Our data are preliminary and support previously obtained results [18, 46], suggesting that the secretion of *Ccl2* by senescent cells correlates with biological age and may be used as a therapeutic target or as a biomarker aimed at monitoring the efficacy of interventions that extend healthy aging. Further studies are necessary to elucidate the mechanisms underlying these alterations.

## MATERIALS AND METHODS

### Animals and experimental design

Genetically modified mice that overexpress *Ccl2* (*CgCcl2*) were generated in our laboratory as described [20]. The mice with the *Lmna ^G609G^* mutation (LA), which constitutes a model of accelerated aging and progeria, were generated at the University of Oviedo [23]. Crossbreeding resulted in the first progeny (F1) of heterozygous animals with the LMNAG609G/+ mutation and *Ccl2* overexpression in tissues (LMNAG609G/+;CGCCL2+/-), which was confirmed by routine genotyping and *Ccl2* measurements (n = 34). In the LMNAG609G/+ mice, the *Ccl2* concentrations in tissues were similar to those observed in the controls, except in adipose tissues, but the *Ccl2* levels in the LMNAG609G/+;CGCCL2+/- mice were significantly higher in all tissues. The second progeny (F2) of the LMNAG609G/+;CGCCL2+/- mice were not fertile, and consequently, homozygotes for the two characteristics were not available. Wild-type (WT) (n = 20) and LMNAG609G/+ mice (n = 28) were used as controls (Supplementary Figure 6). The mice were fed a normal standard diet from Scientific Animal Food & Engineering (SAFE, Augy, France) and water ad libitum for the duration of the experiments and maintained under controlled temperature (22° C), humidity (50%) and a light/dark cycle (12 h/12 h). The animal procedures were approved by the Ethics Review Committee for Animal Experimentation of Universitat Rovira i Virgili (protocols 10281 and FUE-2018-00849209) and designed according to European guidelines (Directive 2010/63/EU).

The mice included in the study were male with a C57BL/6J genetic background, used at 4 weeks of age and assessed daily for lifespan indicators with manipulation maintained at a minimum. To avoid unnecessary suffering and to ensure the lack of bias, we established strict criteria for the decision to sacrifice that was based on a previously reported grading score of senescence (Supplementary Table 3) [47].

### Tissue collection, the preparation of extracts and Ccl2 measurements

All tissues were collected upon mouse sacrifice, and selected pieces were frozen and stored at -80° C until batch analyses. Portions of each 30 mg sample of liver, pancreas, skeletal muscle (from quadriceps muscles), heart, major arteries (aorta and femoral) and brown and epididymal white adipose tissue were homogenized using a sonicator (Branson Sonifer 150, Thistle Scientific, Glasgow, UK) in 50 mM Tris, 1 mM EDTA, 1% IGEPAL CA-630, 150 mM NaCl, 0.10% Triton, 50 mM NaF, 100 mM phenylmethanesulfonyl fluoride (PMS) and 1 mM Na_3_VO_4_. Protein concentration was determined with the Pierce BCA protein assay kit. To measure *Ccl2* by ELISA (PeproTech, London, UK), following the manufacturer’s instructions, 200 μg of protein from each extract was used.

### Histological analysis and immunohistochemistry

Separate portions of the same tissues were collected in formalin (3.7-4% formaldehyde buffered to pH 7 and stabilized with 1-1.5% methanol) and embedded in paraffin. Hematoxylin and eosin and Sirius red staining were used for each tissue. At least 10 sections per mouse were assessed, and the tissue was blindly evaluated to assess defects in structure and detect fibrosis. For immunohistochemistry, antigens were retrieved in Tris 10 mM/EDTA 1 mM buffer at pH 9 in a microwave oven that reached 90° C. Bovine serum albumin (2%) and hydrogen peroxide (1%) were sequentially used to block nonspecific binding sites and endogenous peroxidase, and the sections were rinsed with phosphate-buffered saline. Then, the sections were incubated with primary and secondary antibodies (Supplementary Table 4). All sections were counterstained with Mayer’s hematoxylin and quantified (at least 10 fields for each sample) using ImageJ software.

### Immunoblot analysis

Frozen tissues from each mouse were homogenized in lysis buffer containing 50 mM Tris at pH 7.4, 1 mM EDTA, 1% IGEPAL CA-630, 150 mM NaCl, 0.10% Triton, 50 mM NaF, 100 mM phenylmethanesulfonyl fluoride and 1 mM Na_3_VO_4_. Protein concentration was determined, and the samples were denatured in Laemmli buffer with β-mercaptoethanol at 100° C for 5 min, and 50 μg was loaded per lane onto 8%-14% sodium dodecyl sulfate-polyacrylamide gels. The gels were then transferred to polyvinylidene difluoride or nitrocellulose membranes, blocked with 5% nonfat dry milk or bovine serum albumin in 20 mM Tris at pH 7.4, 150 mM NaCl and 1% Tween-20, and incubated with the respective antibodies (Supplementary Table 4). Immunoreactive bands were developed with a SuperSignal West Femto chemiluminescent substrate (Pierce, Rockford, IL, USA) in a ChemiDoc system (Bio-Rad Laboratories, Madrid, Spain). The bands were quantified using Image Lab 2.0 software (Bio-Rad Laboratories, Hercules, CA, USA).

### Quantitative metabolomics platform and chromatographic procedures

Targeted metabolomics analysis was performed as reported with some modifications [48]. To limit variability, batch analysis was required, and the number of samples measured per day was restricted to n = 24 (n = 8 for each strain). To measure the metabolites of energy metabolism, the tissues were homogenized using a Precellys 24 homogenizer (Izasa, Barcelona, Spain) in 1 mL of methanol/water (8:2) and D4-succinic acid and stored at -20° C for 2 h to precipitate the proteins. After centrifugation at 15,000 rpm for 10 min at 4° C, the supernatants were dried under N_2_ and derivatized with methoxyamine hydrochloride dissolved in pyridine (40 mg/mL) and N-methyl-N-trimethylsilyl trifluoroacetamide. We used a 7890A gas chromatograph coupled with an electron impact source to a 7200 quadrupole time-of-flight mass spectrometer (Agilent Technologies, Santa Clara, USA). For measuring metabolites of 1-C metabolism, tissues were added to 1 mL of an extraction solution containing methanol:water (8:2 v/v), 1% ascorbic acid (m/v) and 0.5% β-mercaptoethanol (v/v) and homogenized with a Precellys 24 homogenizer (Izasa). After protein precipitation, the samples were centrifuged, and the supernatants were dried under N_2_ and then resuspended in ultrapure water containing 50 mM ammonium acetate and 0.2% formic acid. The analysis was performed with an ultra-high pressure liquid chromatography-quadrupole time-of-flight mass spectrometer (Agilent Technologies). Liquid chromatography was also used in separate analyses to quantitate guanine, 5-methylcytosine (5-mC) and 5-hydroxymethylcytosine (5-hmC) as described [49].

### Statistical analyses

The statistical comparisons were performed using nonparametric Mann-Whitney U tests with IBM SPSS Statistics for Windows, Version 21 (Armonk, NY, USA, IBM Corp.). Metabolites were quantitated in all the samples using MassHunter Quantitative Analysis B.07.00 software (Agilent Technologies) according to the calibration curve of the corresponding standard. Partial least squares-discriminant analysis (PLS-DA), hierarchical clustering and the variable importance in the projection score were calculated with MetaboAnalyst 4.0 (http://www.metaboanalyst.ca) software [50]. GraphPad Prism 6.01 software (GraphPad Software, San Diego, CA) was also used for relevant statistical confirmation and graphics. Differences were considered statistically significant when the p value was ≤ 0.05. Unless otherwise indicated, the results are expressed as the means ± standard error of the mean.

## Supplementary Material

Supplementary Figures

Supplementary Tables
