# Supplementary material for: Systemic overexpression of C-C motif chemokine ligand 2 promotes metabolic dysregulation and premature death in mice with accelerated aging
Source: Aging (Albany NY). 2020 Oct 26;12(20):20001–23. doi: 10.18632/aging.104154 (PMC7655213; doi:10.18632/aging.104154)
Supplement: Supplementary Figures [file aging-12-104154-s001..pdf]

Supplementary Figures

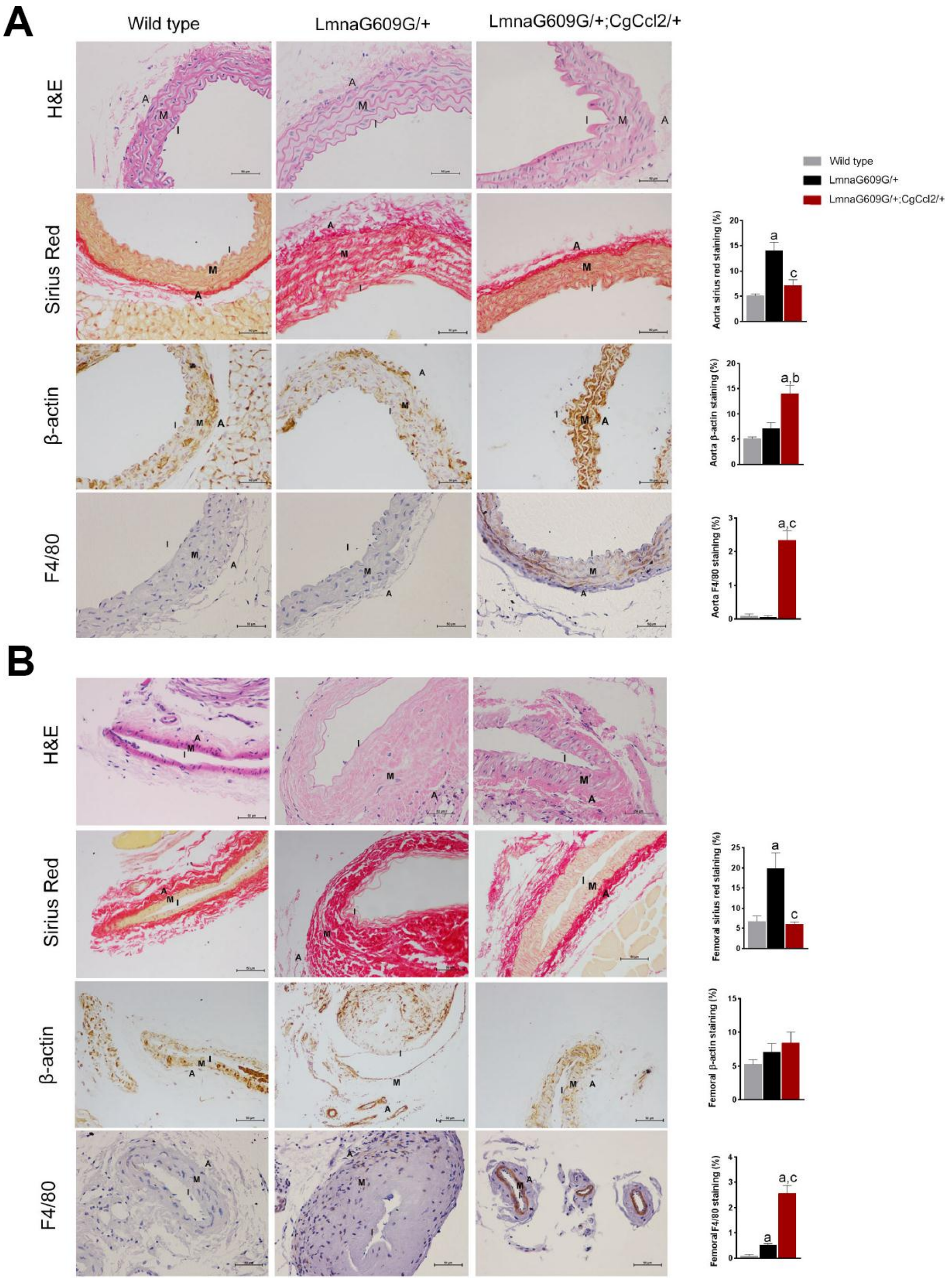

Supplementary Figure 1. Histological alterations in major arteries were accelerated by Ccl2 overexpression. The structure and composition of the aorta (**A**) and femoral arteries (**B**) were clearly disturbed in the progeroid mice with respect to the controls, and Ccl2 exacerbated this negative impact. Representative micrographs taken after staining with hematoxylin and eosin (H&E) and Sirius red and the immunohistochemical analysis of β-actin and F4/80 cells are shown. Results from the measurements of positively stained areas are shown as the means ± SEM. Statistical comparisons of the LMNAG609G/+;Cg CCL2/+ mice are depicted as a $p<0.001$ with respect to the wildtype mice, b $p<0.05$ with respect to the LMNAG609G/+ mice and c $p<0.001$ with respect to the LMNAG609G/+ mice according to the Mann–Whitney U test (n=5 for each strain). LMNAG609G/+ ; Cg CCL2/+ and LMNAG609G/+ denote progeroid mice with and without Ccl2 overexpression, respectively.

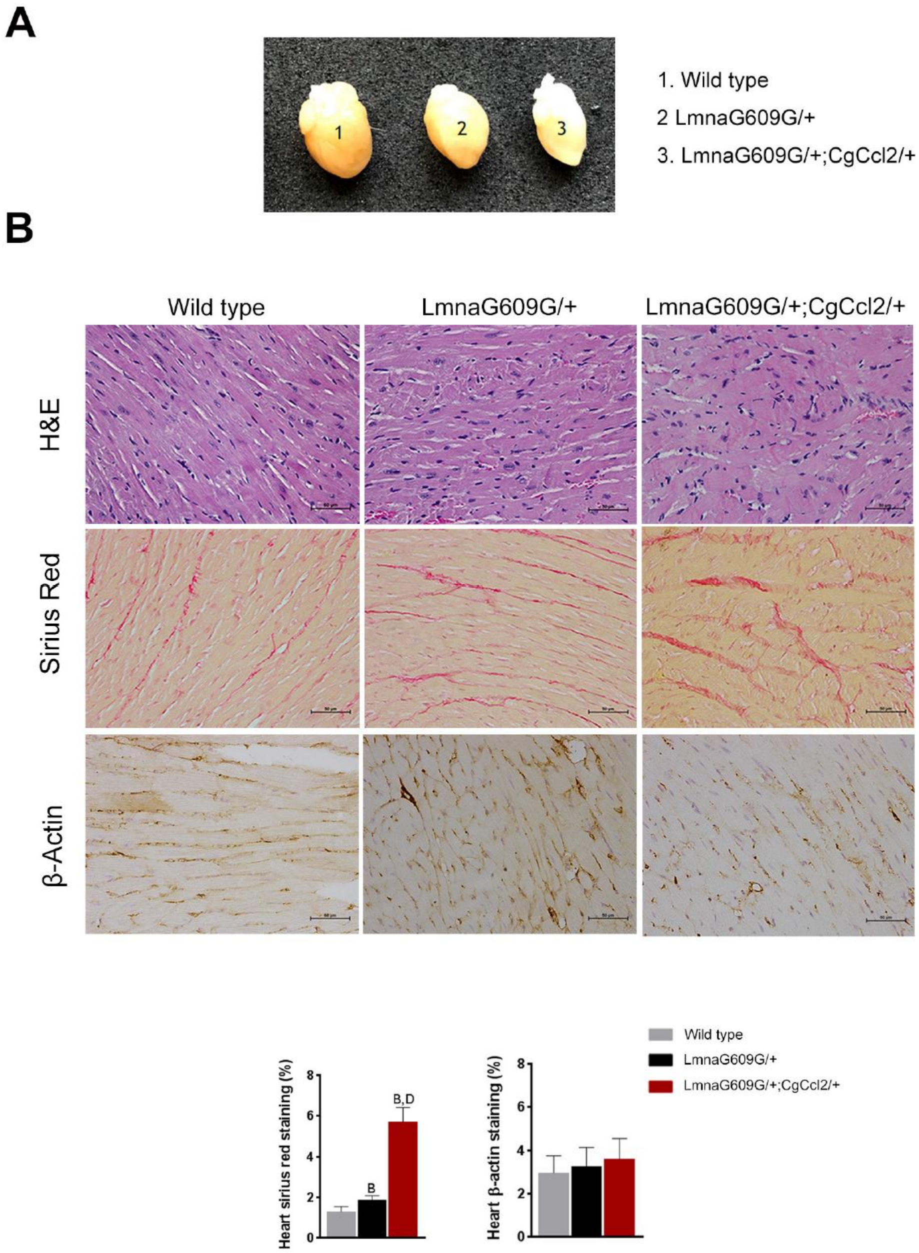

Supplementary Figure 2. Alterations in the histological structure of cardiac muscles of the progeroid mice. (**A**) Representative photograph of animal hearts. (**B**) Representative micrographs of heart sections stained with hematoxylin and eosin (H&E) and Sirius red and results from the immunohistochemical analysis of β-actin showing structural changes, with increased fibrosis being the only feature significantly affected by Ccl2 overexpression. Results from measurements of the positively stained areas are shown as the means ± SEM. Statistical comparisons with the LMNAG609G/+;CGCCL2+/- mice are depicted as a $p<0.05$ with respect to the wild-type mice and b $p<0.05$ with respect to the LMNAG609G/+ mice according to the Mann–Whitney U test (n=5 for each strain). LMNAG609G/+;Cg CCL2/+ and LMNAG609G/+ denote progeroid mice with and without Ccl2 overexpression, respectively.

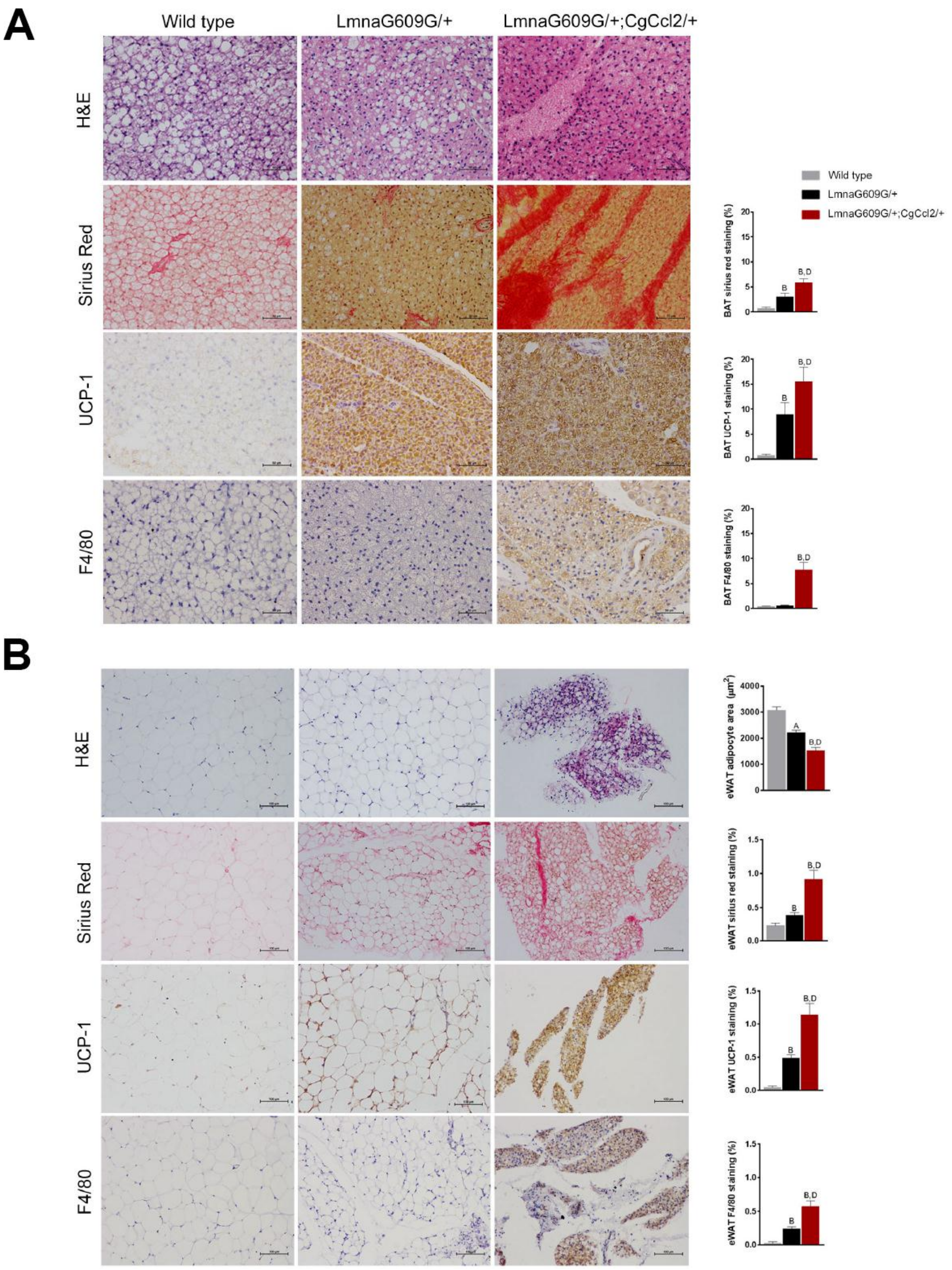

Supplementary Figure 3. The generalized loss of fat depots in the progeroid mice was augmented in the mice with Ccl2 overexpression. Representative micrographs of (**A**) brown (BAT) and (**B**) epididymal white adipose tissue (eWAT) stained with hematoxylin and eosin (H&E) and Sirius red and the results of the immunohistochemical analysis of uncoupling protein one (UCP-1) and F4/80 cells, showing structural changes and the negative impact of Ccl2 overexpression. Results from the measurements of positively stained areas are shown as the means ± SEM. Statistical comparisons with the LMNAG609G/+;Cg CCL2/+ mice are depicted as a $p<0.05$ with respect to the wild-type mice and b $p<0.05$ with respect to the LMNAG609G/+ mice according to the Mann–Whitney U test (n=5 for each strain). LMNAG609G/+;Cg CCL2/+ and LMNAG609G/+ denote progeroid mice with and without Ccl2 overexpression, respectively.

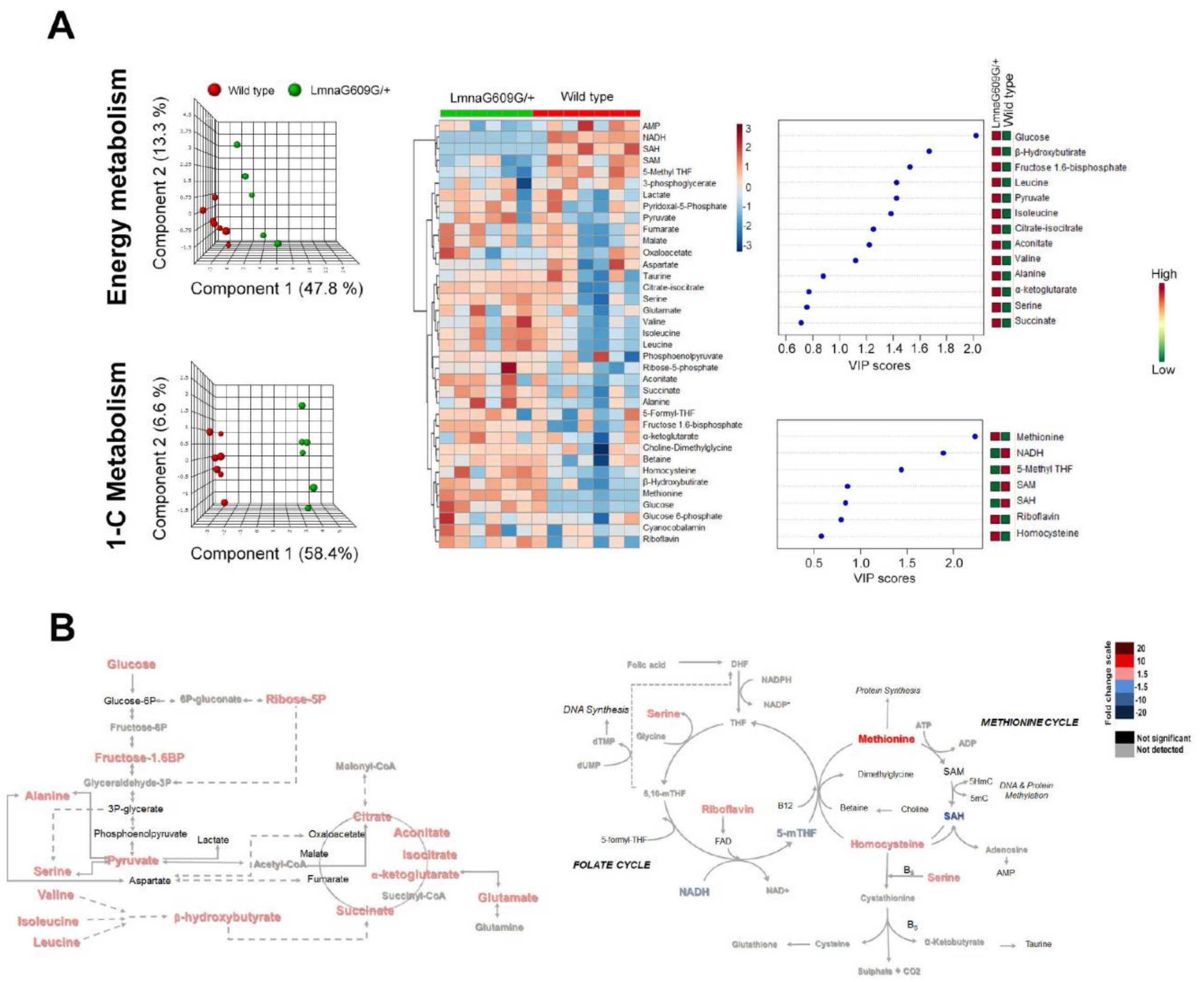

Supplementary Figure 4. Metabolomic analysis of skeletal muscle from LMNAG609G/+ mice and the control group. (**A**) From left to right, partial least square discriminant analysis (PLSDA), heatmap with hierarchical clustering and random forest analysis indicating that Lamin A mutation drove the changes in energy and one-carbon metabolism in the samples from quadriceps muscles. The levels of metabolites distinguish the muscles from this strain from the muscles of the controls (n = 8). The variables of the highest importance, according to the projection scores, were glucose and methionine. (**B**) Comparison of the levels of metabolite abundance from glycolysis and the citric aid cycle in the quadriceps muscles. The relative impact of the Lmna mutation (LMNAG609G/+) was assessed by fold changes, and indicated in the legend, and suggested a failed attempt to increase energy expenditure and attenuate the dysregulation of the methionine cycle.

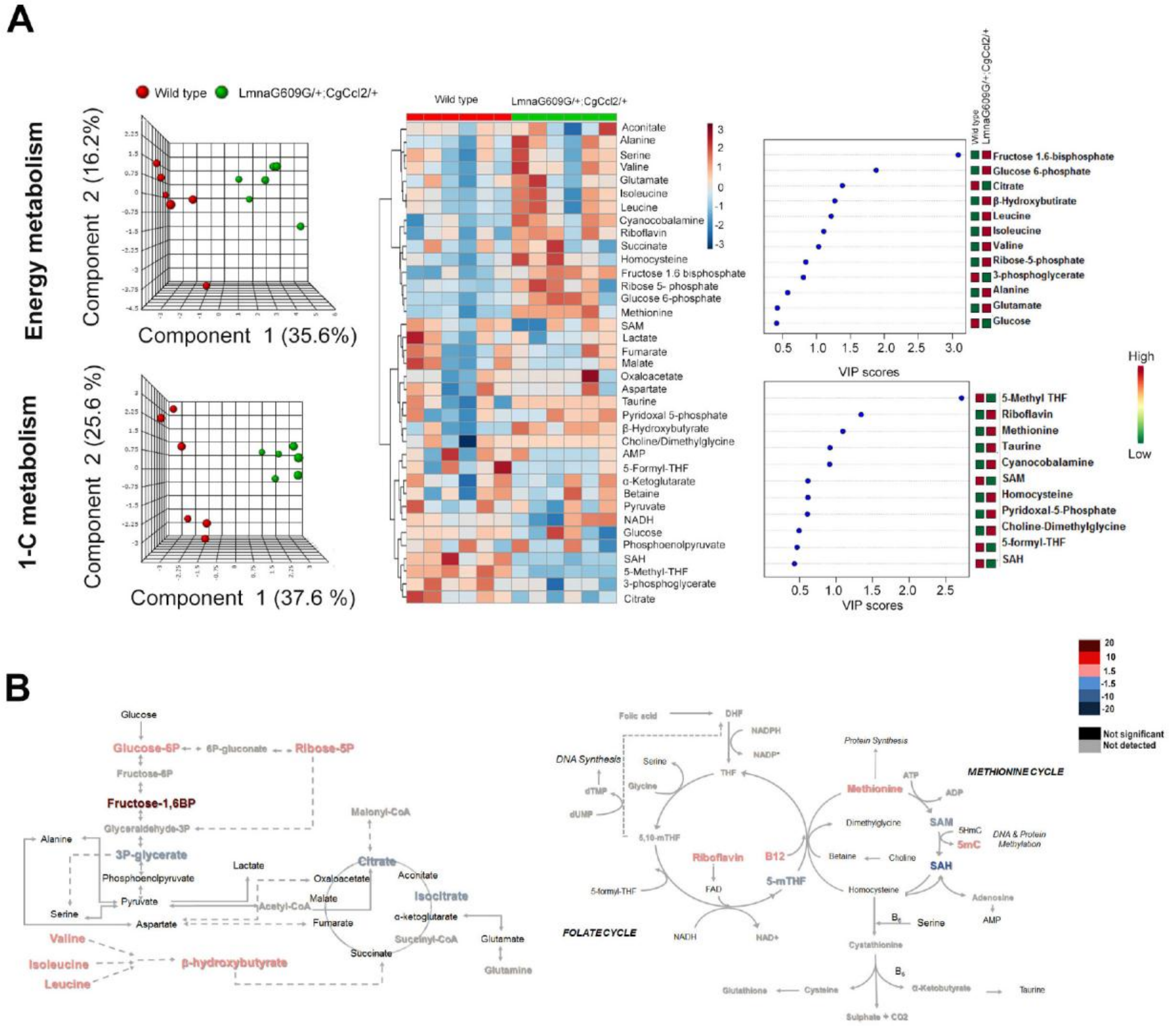

Supplementary Figure 5. Metabolomic analysis of skeletal muscle from LMNAG609G/+;Cg CCL2/+ mice and the control group. (**A**) From left to right, partial least square discriminant analysis (PLSDA), heatmap with hierarchical clustering and random forest analysis indicating that Ccl2 overexpression contributed to the metabolic defects observed in the mice with the simple Lamin A mutation. Consequently, changes in the metabolites of energy and one-carbon metabolism in the samples from quadriceps muscles distinguished the muscles of this strain from the muscles of controls (n = 8). The variables of highest importance, according to the projection scores, were fructose 1,6 biphosphate and 5-methyl tetrahydrofolate (**B**). The relative impact of the LMNAG609G/+;Cg CCL2/+ mutation on the levels of metabolites associated with energy and one-carbon metabolism was assessed by fold changes, as indicated in the legend. The results suggest major defects in the pentose phosphate pathway and folate cycle.

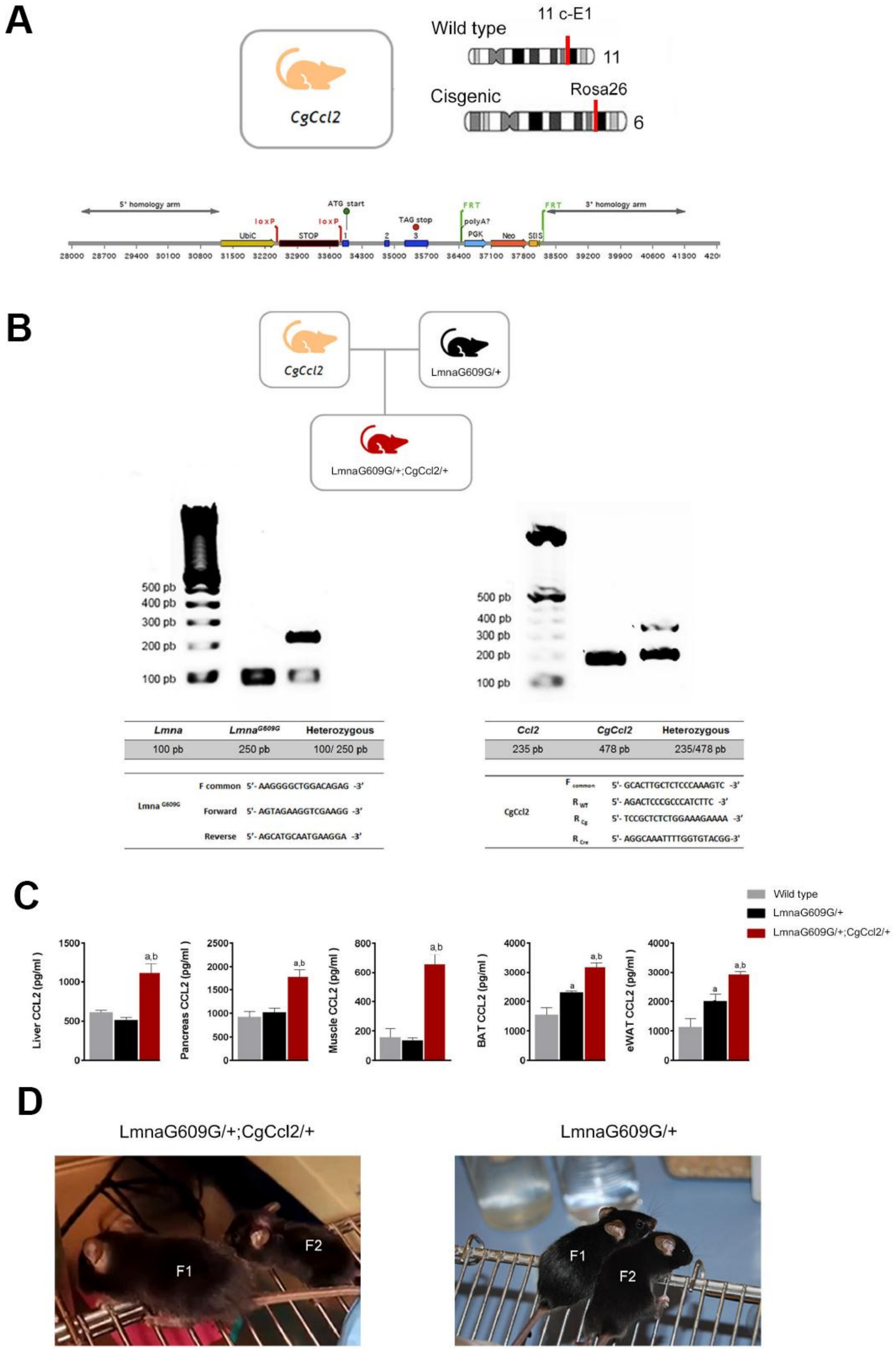

Supplementary Figure 6. Experimental models. (**A**) An additional copy of the C-C motif ligand 2 gene (Ccl2) was inserted via recombination into embryonic stem cells at the ROSA26 locus of chromosome 6 to overexpress Ccl2 [20]. (**B**) Offspring from crossbreeding were systematically genotyped using the described primers to identify the progeroid mice with (LMNAG609G/+;Cg CCL2/+ and without (LMNAG609G/+) [23] Ccl2 overexpression. (**C**) Tissue extracts were used to measure Ccl2 concentration. Results are expressed as the means ± SEM (n = 8-12). Mann–Whitney U tests revealed significant differences denoted as ap< 0.05 with respect to the wild-type mice and bp<0.05 with respect to the LMNAG609G/+ mice. (**D**) F2 littermates of the LMNAG609G/+;Cg CCL2/+ mice were of lower weight and not fertile.
