# Supplementary material for: Systemic overexpression of C-C motif chemokine ligand 2 promotes metabolic dysregulation and premature death in mice with accelerated aging
Source: Aging (Albany NY). 2020 Oct 26;12(20):20001–23. doi: 10.18632/aging.104154 (PMC7655213; doi:10.18632/aging.104154)
Supplement: Supplementary Tables [file aging-12-104154-s002..pdf]

Supplementary Tables

Supplementary Table 1. Energy-balance metabolites in skeletal muscle. Values were expressed in µM/100 mg of tissue (mean and interquartile range).

Metabolites	Wild type	LMNAG609G/+	LMNAG609G/+;Cg CCL2/+
Glucose	373.4 (345-394.2)	1101 (819.4-2248.1)[a]	175.6 (104.5-517.3)[c]
Glucose 6-phosphate	16 (12.8-18.8)	18.2 (14.1-19.7)	131.3 (77.4-166.3) [a,c]
Fructose-1.6-Bisphosphate	3.6 (2.4-13.4)	12.4 (12.4-19.9) [a]	87.9 (55.5-120.4) [a,c]
3-phosphoglycerate	1.9 (1.6-3.2)	1.3 (0.4-2.6)	1.4 (0.8-1.5) [a,c]
Phosphoenolpyruvate	0.5 (0.3-1.1)	0.7 (0.7-0.8)	0.3 (0.1-1.6) [c]
Pyruvate	68.8 (54.8-221.2)	475.6 (248.2-482.6) [a]	107.1 (40-142.4) [c]
Lactate	9815.7 (6626.5-16649.8)	12913.7 (4688.9-16340.4)	9730.5 (8019.7-10986.5)
Ribose-5-phosphate	9 (7.8-9)	10.4 (10.2 -10.7) [a]	20.9 (20.3-29.2) [a,c]
Oxaloacetate	27.7 (15.7-33.4)	19.8 (19.8-38.5)	29.2 (20.7-29.2)
Citrate-isocitrate	51.6 (27.1-119.4)	137.5 (137.5-137.5) [a]	22.8 (11.5-31) [a,d]
Aconitate	11.5 (10.3-12.9)	32.6 (29.4-34.6) [a]	12.7 (7.9-29.4)[c]
α-ketoglutarate	2.1 (1.2-5.1)	3.7 (1.2-5.2) [a]	1.6 (1.2-5.2)
Succinate	41.2 (38.1-79.1)	71.1 (66.5-104.5)[a]	47.8 (25.4-83.5)
Fumarate	143.7 (56.9-217.6)	337.4 (229.7-337.4)	131.9 (89.1-160.5)[c]
Malate	322.2 (133.8-502)	593 (387.8-704.6)	231 (203.7-326.1)[c]
β-Hydroxybutirate	38.4 (22-51.3)	129.9 (97.6-147.4) [a]	99.5 (82.9-111.9) [a]
Glutamate	1673 (1212.6-2498.8)	2798.1 (1842.4-3290.2) [a]	2058.4 (1558.2-3866.8)
Alanine	362.2 (286.4-414.6)	619.2 (396.8-1193.4) [a]	489.4 (342.4-855.5)
Serine	1007.8 (591.2-1375)	1585.4 (1360-2012.6) [a]	1309.3 (956.5-1466)
Valine	123.3 (75.9-201.2)	357.5 (164-814.4) [a]	258 (220.7-507.8) [a]
Isoleucine	77.1 (45-116.7)	317.9 (126.3-341.8) [a]	174.6 (101.1-438.8) [a]
Leucine	163.8 (102-260.6)	612.9 (287-851.5) [a]	416 (267.4-1153.4) [a]
Aspartate	245 (108.4-374.3)	195.5 (195.5-201.5)	206.3 (139-219.3)

[a] $p < 0.05$, [b] $p < 0.001$, with respect to wild type; [c] $p<0.05$, [d] $p<0.001$, with respect to LMNAG609G/+ by Mann–Whitney U test. *LMNAG609G/+*;Cg CCL2/+ *andLMNAG609G*/+ denote progeroid mice with and without Ccl2, respectively.

Supplementary Table 2. Metabolites from one-carbon metabolism in skeletal muscle.

Metabolites	Wild type	LMNAG609G/+	LMNAG609G/+;CgCCL2/+
Taurine	1499.7 (601.8-3621.9	1952.7 (1407-2041.3)	3038.1 (2920.8-3038.1)[d]
Choline-Dimethylglycine*	145.9 (69.1-216.1)	185.7 (185.7-189.4)	171.6 (171.0-171.8)[d]
Betaine*	0.4 (0.2-0.5)	0.6 (0.5-0.6)	0.3 (0.3-0.6)
Homocysteine	0.3 (0.3-0.4)	0.7 (0.5-0.9)[a]	0.5 (0.3-0.9)
SAM	3.3 (1.7-4.5)	1.2 (0.7-2.0)	2.7 (0.5-2.1)[a,c]
Methionine	0.6 (0.6-0.7)	9.3 (6.4-12.2)[a]	1.6 (0.8-1.6)[a,d]
AMP	3.7 (2.1-5.7)	2.3 (1.5-2.9)	2.8 (2.8-3.8)[c]
NADH	1.7 (1.3-2.5)	0.2 (0.2-0.2)[a]	5 (0.2-3.8)[d]
SAH*	0.1 (0.1-0.2)	0.01 (0.0-0.01)[a]	0.09 (0.07-0.1)[b,d]
Pyridoxal 5 phosphate	3.5 (2.2-7.1)	4.9 (4.4-6.4)	6.7 (4.1-9.6)
5-Methyl-THF*	10.2 (8.8-20.1)	3.2 (1.2-3.2)[a]	1.1 (0.9-1.3)[a,c]
5-Formyl-THF*	1.7 (0.7-2.8)	2.1 (2.1-2.1)	0.8 (0.8-1.3)[c]
Cyanocobalamin*	0.4 (0.3-0.5)	0.5 (0.4-0.8)	0.8 (0.5-1)[a]
Riboflavin	0.5 (0.2-0.9)	1.2 (0.9-1.7)[b]	1.7 (0.9-2.2)[a]

Values were expressed in µM/ 100 mg of tissue (mean and interquartile range) or * nM/100 mg of tissue. [a] $p < 0.05$, [b] $p < 0.001$, with respect to wild type; [c] $p < 0.05$, [d] $p < 0.001$, with respect to LMNAG609G/+ by Mann–Whitney U test. LMNAG609G/+*;CgCCL2/+* and LMNAG609G/+ denote progeroid mice with and without *Ccl2*, respectively. SAM: s-adenosylmethionine; AMP: Adenosine monophosphate; SAH: s-adenosylhomocysteine; 5-Methyl-THF: 5-Methyl-tetrahydrofolate; 5-Formyl-THF: 5-Formyl-tetrahydrofolate.

Supplementary Table 3. Criteria for grading score of senescence in mice.

		Grade 0	Grade 1	Grade 2	Grade 3	Grade 4
**Behavior**						
**Reactivity**	The most intensive exploratory response observed within 30 seconds	Normal behavior	Abnormal grait with no lessening of agility and behavior patterns and restlessness	Definite decrease in agility and behavior patterns	Does not move voluntarily but will move if nudged	Immobile
**Passivity**	Escape reaction from pinching of the nuchal skin or from hanging by the forelimb	Natural escape reaction to pinching	Decrease in escape reaction to pinching	Loss of escape reaction to pinching. Preserved righting reaction to manual turn over	Neither escape reaction to pinching nor righting to hanging by the forelimb.	Escape reaction nil
**Appearance**						
**Glossiness**	Glossiness	Natural gloss	Decrease in gloss	Complete disappearance of gloss	Complete disappearance of gloss and hair appears dirty	Complete disappearance of gloss and hair looks very dirty
**Coarseness**	Coarseness of hair on the head, nuca and dorsum determined according to the number of palpable, fine clumps of hair	No coarseness	Coarseness of less than an area of the head	Coarseness of less than double the area of the head	Coarseness of less than 3 times area of the head	Coarseness of over 3 times area of the head
**Loss of hair**	Loss or thinning of hair on the head, nuca and dorsum except for changes due to ulcer or Periophthalmic lesions	Neither loss or thinning of hair	A. Loss of hair in less than an area of the head. B. Thinning of hair in less than 1/2 of total area	A. Loss of hair in over one area of the head, less than in 1/4 of total area. B. Thinning if hair in more than 1/2 of total area	Loss of hair in more than 1/4, in less than 1/2 of total area.	Loss of hair in over 1/2 of total area
**Skin ulcers**	Ulcer or healed ulcer on entire skin except for changes associated with Periophthalmic lesions	No evidence of ulcer	Healed ulcer or ulcer with scab	Ulcer without healing tendency, in less than one area of the head	Ulcer without healing tendency in more than one area of the heard, in less than 1/4 area of all the skin.	Ulcer without healing tendency in more than 1/4 area of whole skin
**Eyes**						
**Periophthalmic lesions**	Catarrhal changes in the periophthalmic area or swelling of the palpebra	No changes	Catarrhal changes limited to periophthalmic area or swelling of palpebra	Catarrhal changes extending to nose	Catarrhal changes extending further	
**Lordokyphosis of the spine**	Examined by inspection and palpation	Natural anteroposterior curvature	Increased curvature disappears with digital pressure on the dorsum	Increased curvature disappears with a combination of manual cephalocaudal traction and digital pressure on the dorsum	permanent curvature	Lordokyphosis of the spine

Supplementary Table 4. Antibodies used in western blot and immunohistochemical analyses.

Antigen	Antibody	1ry Dilution	2ry Antibody	2ry Dilution
CD11b	CD11b antibody, ab133357 (Abcam, Cambridge, UK)	1:1000	Goat α-rabbit HRP, P0448 (Dako, Santa Clara, CA, USA)	1:5000
TNFα	TNFα antibody, #3707 (Cell signalling, Danvers, MA, USA)	1:1000	Goat α-rabbit HRP, P0448 (Dako, Santa Clara, CA, USA)	1:5000
CD163	CD163, antibody, ab182422 (Abcam, Cambridge, UK)	1:1000	Goat α-rabbit HRP, P0448 (Dako, Santa Clara, CA, USA)	1:5000
F4/80	F4/80, antibody, ab100790 (Abcam, Cambridge, UK)	1:100	Goat α-rabbit HRP, P0448 (Dako, Santa Clara, CA, USA)	1:200
Arginase	Liver arginase, antibody, ab91279 (Abcam, Cambridge, UK)	1:10000	Goat α-rabbit HRP, P0448 (Dako, Santa Clara, CA, USA)	1:5000
PON1	In-house	1:200	Goat α-rabbit HRP, P0448 (Dako, Santa Clara, CA, USA)	1:1000
PI3K-p85	PI3K antibody, #4257 (Cell signalling, Danvers, MA, USA)	1:1000	Goat α-rabbit HRP, P0448 (Dako, Santa Clara, CA, USA)	1:5000
Akt pS473	p-Akt antibody, #4060 (Cell signalling, Danvers, MA, USA)	1:1000	Goat α-rabbit HRP, P0448 (Dako, Santa Clara, CA, USA)	1:5000
Akt	AKT antibody, #4685 (Cell signalling, Danvers, MA, USA)	1:1000	Goat α-rabbit HRP, P0448 (Dako, Santa Clara, CA, USA)	1:5000
S6 pS235/236	p-pS6 antibody, #4856 (Cell signalling, Danvers, MA, USA)	1:1000	Goat α-rabbit HRP, P0448 (Dako, Santa Clara, CA, USA)	1:5000
S6	S6 antibody, #2217 (Cell signalling, Danvers, MA, USA)	1:1000	Goat α-rabbit HRP, P0448 (Dako, Santa Clara, CA, USA)	1:5000
P53 p-S15	P-P53 antibody, #9284 (Cell signalling, Danvers, MA, USA)	1:1000	Goat α-rabbit HRP, P0448 (Dako, Santa Clara, CA, USA)	1:500
P53	P53 antibody, #2524 (Cell signalling, Danvers, MA, USA)	1:1000	Goat α-mouse HRP, P0447 (Dako, Santa Clara, CA, USA)	1:500
AMPK pT172	p-AMPK antibody, #2531 (Cell signalling, Danvers, MA, USA)	1:1000	Goat α-rabbit HRP, P0448 (Dako, Santa Clara, CA, USA)	1:5000
AMPK	AMPK antibody #2532S (Cell signalling, Danvers, MA, USA)	1:1000	Goat α-rabbit HRP, P0448 (Dako, Santa Clara, CA, USA)	1:5000
OXPHOS	OXPHOS Rodent,antibody, ab ab110413 (Abcam, Cambridge, UK)	1:250	Goat α-mouse HRP, P0447 (Dako, Santa Clara, CA, USA)	1:5000
LC3	LC3B Antibody, #2775S (Cell signalling, Danvers, MA, USA)	1:1000	Goat α-rabbit HRP, P0448 (Dako, Santa Clara, CA, USA)	1:5000
LAMP2A	LAMP2A antibody, ab125068 (Abcam, Cambridge, UK)	1:1000	Goat α-rabbit HRP, P0448 (Dako, Santa Clara, CA, USA)	1:2000
P62	P62 Antibody, #5114 (Cell signalling, Danvers, MA, USA)	1:1000	Goat α-rabbit HRP, P0448 (Dako, Santa Clara, CA, USA)	1:5000
Vinculin	Vinculin antibody, ab73412 (Abcam, Cambridge, UK)	1:1000	Goat α-rabbit HRP, P0448 (Dako, Santa Clara, CA, USA)	1:2000
TOM20	TOM20 antibody, #42406 (Cell signalling, Danvers, MA, USA)	1:1000	Goat α-rabbit HRP, P0448 (Dako, Santa Clara, CA, USA)	1:5000
Mfn2	Mfn2 antibody, ab124773 (Abcam, Cambridge, UK)	1:1000	Goat α-rabbit HRP, P0448 (Dako, Santa Clara, CA, USA)	1:2000
UCP-1	UCP-1, antibody, ab10983(Abcam, Cambridge, UK)	1:100	Goat α-rabbit HRP, P0448 (Dako, Santa Clara, CA, USA)	1:200
